# Epidemiological and clinical profiles of acute poisoning in patients admitted to the intensive care unit in eastern Iran (2010 to 2017)

**DOI:** 10.1186/s12873-018-0181-6

**Published:** 2018-09-19

**Authors:** Omid Mehrpour, Ayob Akbari, Firoozeh Jahani, Alireza Amirabadizadeh, Elaheh Allahyari, Borhan Mansouri, Patrick C. Ng

**Affiliations:** 1 Medical Toxicology and Drug Abuse Research Center (MTDRC), Birjand University of Medical Sciences, Moallem Avenue, Birjand, 9717853577 Iran; 2 Rocky Mountain Poison and Drug Center, Denver, CO USA; 3 Social Determinants of Health Research Center, Birjand University of Medical Sciences, Birjand, Iran

**Keywords:** Acute poisoning, Intensive care unit (ICU), Opioids, Benzodiazepines, Pesticides

## Abstract

**Background:**

Acute poisoning is a common chief complaint leading to emergency department visits and hospital admissions in developing countries such as Iran. Data describing the epidemiology of different poisonings, characteristics of the clinical presentations, and the predictors of outcome are lacking. Such data can help develop more efficient preventative and management strategies to decrease morbidity and mortality related to these poisonings. This manuscript describes the epidemiology of acute poisoning among patients admitted to the intensive care unit (ICU) in Birjand, Iran.

**Methods:**

This retrospective, cross-sectional study was conducted to characterize acute poisonings managed in the ICU during a 7-year period from March 2010 to March 2017 in a single center in Birjand, Iran. Patient characteristics, suspected exposure, the route of exposure, and outcome data were collected from hospital medical records.

**Results:**

During the study period, 267 (64% male and 36% female) patients met inclusion criteria. Pharmaceutical medication (36.6%), opioids (26.2%) followed by pesticides (13.9%) were the most common exposures 38.2% of these cases were identified as suicide attempts. There were different frequencies in terms of xenobiotic exposure in relation to gender (*p* = 0.04) and the survival (*p* = 0.001). There was a significant difference between various xenobiotics identified as the cause of poisoning (p = 0.001). Mortality rate in our study was 19.5%. The incidence of outcomes was significantly higher in patients poisoned with opioids, pesticides, benzodiazepines, and tricyclic antidepressants (*p* < 0.05). The median length of hospital stay was higher in pesticide-poisoned patients (*p* = 0.04).

**Conclusion:**

Opioids and pesticides were the most common exposures. The mortality rate of the poisoned patients in the ICU was proportionately high. The mortality rate due to opioid poisoning is a major concern and the most significant cause death due to poisoning in the region. Further monitoring and characterization of acute poisoning in Birjand, Iran is needed. These data can help develop educational and preventative programs to reduce these exposures and improve management of exposures in the prehospital and hospital settings.

## Background

A poison is a xenobiotic that, in the right dose, can lead to injury or death of an organism [1]. Poisoning is a common, resource intensive chief complaint resulting in thousands of hospital admissions worldwide. Acute poisoning, depending on the xenobiotic, can present in many ways. Some signs and symptoms associated with poisoning include: CNS depression, miosis, hypothermia, respiratory depression, hypotension, delirium, dysrhythmias and multisystem organ failure [2–4]. Poisoning can be categorized into intentional and accidental. Many cases of intentional poisoning occur in developing countries where resources are limited and are associated with a high degree of morbidity and mortality [3, 5, 6].

The prevalence of acute poisoning varies in relation to religious, cultural, and geographical contexts and is dynamic given the continued development and varying availability of different xenobiotics [4]. For example, in developed countries the most common cause of acute poisoning is the abuse of commercially available pharmaceuticals [7], in contrast, in developing countries, insecticides are the most common [8]. Furthermore, the availability of pharmaceuticals is regional; in Iran, prescriptions for and availability of commercially available opioids are significantly less compared to countries like the United States. In Iran, the majority of poisonings are intentional and occur mainly in the age range of 21–30 years. In this country, the mortality rate from poisoning is 8 per 1000 individuals in the general hospitalization wards and 109 per 1000 people in the intensive care unit (ICU). According to the World Health Organization (WHO), suicide and chemical substances account for nearly one million deaths annually worldwide with pesticides as a major cause [9]. Timely diagnosis of poisoning and appropriate treatment is vital to prevent morbidity and mortality.

More data on the general pattern of poisonings in each geographic region is important to address this issue. To reduce hospital morbidity and mortality, early diagnosis and rapid treatment in ED and ICU are critical for the poisoned patient. Currently, there are very few data available that detail poisonings in Eastern Iran especially in South Khorasan province. Despite this need, few studies in Iran have addressed the patterns of poisoning in the patients hospitalized in the ICU [9]. The main aim of this study was to assess the epidemiological characteristics and clinical features of acute poisoning in all adult patients admitted to the Imam Reza Hospital ICU in Birjand City, Iran from 2010 to 2017. With a deeper understanding of poisoning in Iran, more effective education and management plans can lead to more efficient recognition and management of these patients, eventually reducing morbidity and mortality associated with this potentially deadly chief complaint.

## Methods

### Study design

**Fig. 1:**
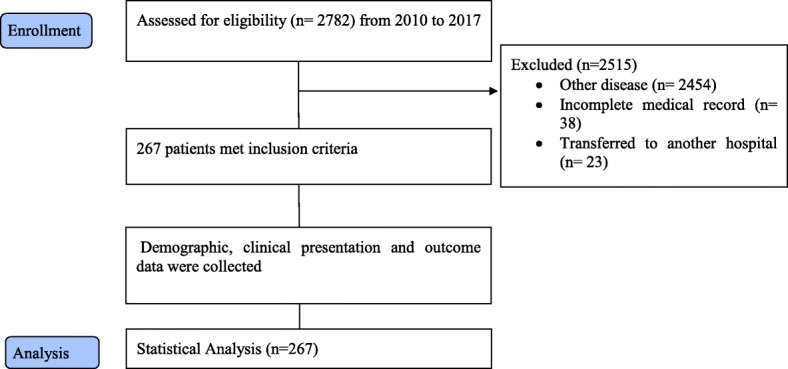
Flow chart of study patient selection

This retrospective, observational cross-sectional study was conducted to investigate the clinical characteristics and epidemiological patterns of acute poisoning leading to ICU admission. The study took place in Imam Reza Hospital in Birjand City, South Khorasan Province, Iran from March 21, 2010 to March 21, 2017 (Fig. 1). The study protocol was approved by a local institutional review board. Imam Reza Hospital, a teaching hospital affiliated with Birjand University of Medical Sciences, is the referral center for toxicology patients in the South Khorasan Province [10].

### Study population

The study population consisted of all individuals diagnosed with acute poisoning admitted to the ICU of Imam Reza Hospital in Birjand City during the seven years. The inclusion criteria were: Age > 13 years and chief complaint of acute poisoning. In our center, patients less than 13 years old are usually admitted to a pediatric unit. Exclusion criteria included: Age < 13 years, history of underlying chronic disease (e.g. diabetes mellitus and renal failure), presentation with concomitant acute pathology with intoxication (such as burns, trauma, etc.), and/or incomplete medical file.

### Data gathering

Poisoned patients in the emergency department were examined and treated by the physicians on duty. Depending on the severity of symptoms, the patients were discharged after management in the emergency department, transferred to an 18-bed specialty poisoning ward, or admitted to the ICU. ICU admission decisions were made by the ICU physician on duty. Criteria for admission to the ICU included: potentially lethal exposure (for example, aluminum phosphide (AlP), strychnine, arsenic, or cyanide), seizure, deep CNS depression, respiratory distress (RR > 35 breaths/min), and hemodynamic instability (systolic arterial pressure < 80 mmHg or 20 mmHg below the patient’s usual pressure) [11]. A standardized data collection sheet was used to collect demographic characteristics (age, gender, etc.), timing of presentation, the cause of poisoning (accidental, overdose, intentional), the suspected exposure (envenomation, asphyxiates, opioids (methadone, heroin, opium, opium residue), insecticides, and pharmaceutical drugs), the outcome (in-hospital mortality, discharge) and vital signs. Data was collected from patient medical records. This study was approved by the ethics committee of Birjand University of Medical Sciences with ethics code of IR.BUMS.1395.6. Administrative permissions were obtained, in order to review patient records from ethics committee of Birjand University of Medical Sciences.

### Statistical analysis

Data analysis was performed by the SPSS package, version 22 (Chicago, IL, USA). Descriptive statistics including frequency, percentage, mean, and standard deviation were reported. Using Kolmogorov-Smirnov test, we examined the normality of quantitative variables distribution. Mann-Whitney and Kruskal-Wallis tests were employed to analyze quantitative variables with non-normal distribution. Chi-square and Fisher’s exact tests were used to analyze qualitative data. The significance level was considered to be 0.05.

## Results

During the study period, 267 of 2782 cases reviewed met inclusion criteria, 173 (64.8%) of which were male. Most of the cases [*n* = 72; 27%] occurred in summer.

Pharmaceutical drug poisoning was the most common cause of intoxication (36.6%). Of these, benzodiazepines were the most frequent, followed by tricyclic antidepressants. Opioids (26.2%) (Methadone, opium, opium residue) and Pesticide (13.9%) were also common exposures (Table 1).

**Table 1:** Frequency distribution of demographic variables in the studied patients

Variable		Number of cases (n)	Percentage (%)
Gender	Male	173.0	64.8
	Female	94.0	35.2
Age group (years)	20–35	167.0	62.5
	35–50	46.0	17.2
	50–65	26.0	9.5
	> = 65	28.0	10.5
Residence	Urban	183.0	69.6
	Rural	84.0	30.4
Education status	Illiterate	25.0	9.4
	Primary school	26.0	9.7
	Secondary school	43.0	16.1
	High school	32.0	11.9
	College	28.0	10.5
	Unknown	113.0	42.4
Employment status	Student	13.0	4.8
	Unemployed	68.0	25.5
	Employed	26.0	9.7
	Farmer	14.0	5.2
	Others	45.0	16.8
	Unknown	101	38.0
Season	Spring	62.0	23.2
	Summer	72.0	27.0
	Autumn	63.0	23.6
	Winter	70.0	26.2
Glasgow Coma Scale (GCS)	< 9	124.0	46.4
	9–13	51.0	19.1
	13–15	92.0	34.5

There was a difference in terms of the exposure identified between men and women (χ^2^ = 23.25, *p* = 0.04). Of the deceased patients, 36 (20.8%) were men and 16 (17.1%) were women. The data did not detect a significant difference in outcomes between men and women (*p* = 0.45).

One hundred two (38.2%) [Male = 60] of cases were related to suicide. Twenty two (8.2%) cases were identified as accidental poisonings. There was no significant different between male and female regarding cause of poisoning (*p* = 0.16) (Table 2). There was a significant difference between various xenobiotics identified as the cause of poisoning (*p* = 0.001), Table 3.

**Table 2:** Comparison of the frequencies of poisoning agents, route of use, causes of poisoning, and poisoning outcomes per gender

Variable	Total	Male	Female	Test result
Poisoning agent				χ^2^ = 23.25 *P* =0.04
Acetaminophen	2 (0.7%)	2 (1.2%)	0 (0%)	
Tramadol	9 (3.4%)	5 (2.9%)	4 (4.3%)	
Benzodiazepines	34 (12.7%)	22 (12.7%)	12 (12.8%)	
Tricyclic antidepressants	23 (8.6%)	11 (6.4%)	12 (12.8%)	
Antipsychotics	20 (7.5%)	11 (6.4%)	9 (9.6%)	
Anticonvulsant	4 (1.5%)	4 (2.3%)	0 (0%)	
Beta Blocker	6 (2.2%)	5 (2.9%)	1 (1.1%)	
Opioids	70 (26.2%)	53 (30.6%)	17 (18.1%)	
Alcohol	4 (1.5%)	4 (2.3%)	0 (0%)	
Cannabis	3 (1.1%)	3 (1.7%)	0 (0%)	
Co-Poisoning	8 (3.0%)	5 (2.9%)	3 (3.2%)	
Aluminum phosphide	2 (0.7%)	1 (0.6%)	1 (1.1%)	
Pesticides	37 (13.9%)	19 (11.0%)	18 (19.1%)	
Others	11 (4.1%)	4 (2.3%)	7 (7.4%)	
Unknown	34 (12.7%)	24 (13.9%)	10 (10.6%)	
total	267 (100%)	173(100%)	94 (100%)	
Cause of poisoning				χ^2^ = 5.09 *P* = 0.16
Accidental	22 (8.2%)	12 (6.9%)	10 (10.6%)	
Suicide	102 (38.2%)	60 (34.7%)	42 (44.7%)	
Overdose	49 (18.4%)	33 (19.1%)	16 (17.0%)	
Unknown	94 (35.2%)	68 (39.3%)	26 (27.7%)	
Route of use				
Oral ingestion	215 (80.5%)	137 (79.2%)	78 (83.0%)	χ^2^ = 0.77 *P* = 0.86
Inhalation	11 (4.1%)	7 (4.0%)	4 (4.3%)	
Injection	3 (1.1%)	2 (1.2%)	1 (1.1%)	
Unknown	38 (14.2%)	27 (15.6%)	11 (11.7%)	
Outcome				χ^2^ = 0.56 *P* = 0.45
Survival	215 (80.5%)	137 (79.2%)	78 (83.0%)	
Death	52 (19.5%)	36 (20.8%)	16 (17.0%)

**Table 3:** Frequency of poisoning cases according to cause of poisoning

Poisoning agent	Accidental	Suicide	Overdose	Unknown	Test results
Acetaminophen	0(0%)	2(100%)	0(0%)	0(0%)	χ^2^ = 219.03 *P* < 0.001
Tramadol	0(0%)	6(66.7%)	0(0%)	3(33.3%)	
Benzodiazepines	1(2.9%)	20(58.8%)	1(2.9%)	12(35.3%)	
Tricyclic antidepressants	0(0%)	16(69.6%)	0(0%)	7(30.4%)	
Antipsychotics	0(0%)	17(85%)	0(0%)	3(15%)	
Anticonvulsant	0(0%)	3(75%)	0(0%)	1(25%)	
Beta Blocker	0(0%)	4(66.7%)	0(0%)	2(33.3%)	
Opioids	6(8.6%)	7(10%)	43(61.4%)	14(20%)	
Alcohol	0(0%)	2(50%)	2(50%)	0(0%)	
Cannabis	0(0%)	0(0%)	0(0%)	3(100%)	
Co-Poisoning	2(25%)	0(0%)	0(0%)	6(75%)	
Aluminum phosphide	0(0%)	2(100%)	0(0%)	0(0%)	
Pesticides	9(24.3%)	14(37.8%)	0(0%)	14(37.8%)	
Others	3(27.3%)	2(18.2%)	3(27.3%)	3(27.3%)	
Unknown	1(2.9%)	7(20.6%)	0(0%)	26(76.5%)	

Seventy (26%) of cases were opioid related (Table 4). The Bonferroni test showed that the incidence of outcomes was significantly higher in patients poisoned with opioids, pesticides, benzodiazepines, tricyclic antidepressants (TCAs), and unspecified agents than in those poisoned with other medications and substances (*p* < 0.05). According to the results of this study, 52 (19.5%) of the patients admitted to the ICU died. One-hundred and ten patients (41.2%) were intubated, of whom 47 (17.6%) died and 63 (23.6%) survived. Among those discharged (*n* = 215), 12 patients left the hospital before completion of treatment and discharged against medical advice.

**Table 4:** Poison exposure for survivors and non-survivors of poisoning admitted to Intensive Care Unit (ICU)

		Total	Survival	Non-survivors	Test result
poisoning agents	Acetaminophen	2 (0.7%)	2 (0.9%)	0 (0%)	χ2 = 37.08 *P* = 0.001
	Tramadol	9 (3.4%)	8 (3.7%)	1 (1.9%)	
	Benzodiazepines	34 (12.7%)	32 (14.9%)	2 (3.8%)	
	Tricyclic antidepressants	23 (8.6%)	21 (9.8%)	2 (3.8%)	
	Antipsychotics	20 (7.5%)	20 (9.3%)	0 (0%)	
	Anticonvulsant	4 (1.5%)	4 (1.9%)	0 (0%)	
	Beta Blocker	6 (2.2%)	6 (2.8%)	0 (0%)	
	Opioids	70 (26.2%)	45 (20.9%)	25 (48.1%)	
	Alcohol	4 (1.5%)	2 (0.9%)	2 (3.8%)	
	Cannabis	3 (1.1%)	3 (1.4%)	0 (0%)	
	Co-Poisoning	8 (3.0%)	7 (3.3%)	1 (1.9%)	
	Aluminum phosphide	2 (0.7%)	2 (0.9%)	0 (0%)	
	Pesticides	37 (13.9%)	33 (15.3%)	4 (7.7%)	
	Others	11 (4.1%)	8 (3.7%)	3 (5.8%)	
	Unknown	34 (12.7%)	22 (10.2%)	12 (23.1%)	
	total	267 (100%)	215 (100%)	52(100%)	
Intubation	Yes	110.0 (41.2%)	63 (23.6%)	47 (17.6%)	χ2 = 64.49 *p* < 0.001
	No	157 (58.8%)	152 (56.9%)	5 (1.9%)	
Seizure	Yes	33 (12.4%)	24 (9.0%)	9 (3.4%)	χ2 = 1.43 *P* = 0.17
	No	234 (87.6%)	191 (71.4%)	43 (16.2%)	

Table 5 shows median length of hospital stay in various types of poisoning. Kruskal-Wallis testing determined that the median length of hospital stay was significantly higher in the patients treated for pesticide poisoning than in those poisoned patients treated for poisoning from other xenobiotics (χ2 = 23.89, *p* = 0.04). The median duration of hospital stay in the orally poisoned patients was 3.0 [2.0–4.0] days with the mean duration of 4.66 ± 6.74 days. In overdose patients, the median length of hospital stay was 3.0 [2.0–5.5] days with the mean duration of 4.94 ± 6.04 days. The median length of hospital stay in the deceased patients was 5.5 [2.0–13.0] days with the mean duration of 8.53 ± 8.99 days. The results of the Mann-Whitney test showed that the median length of hospital stay was significantly higher in the deceased patients than in the survived ones (z = 4.27, *p* < 0.001).

**Table 5:** Comparison of median length of hospital stay among study population

Variable		Length of hospital stay (days) Median[IQR]	Test result
Poisoning agent	Acetaminophen	2.0 [1.0–2.0]	χ2 = 23.89 *P* = 0.04
	Tramadol	2.0 [1.5–3.5]	
	Benzodiazepines	2.0 [1.0–3.0]	
	Tricyclic antidepressants	2.0 [2.0–3.0]	
	Antipsychotics	2.0 [1.25–3.0]	
	Anticonvulsant	3.0 [2.0–4.0]	
	Beta Blocker	2.5 [1.0–3.0]	
	Opioids	3.0 [2.0–6.0]	
	Alcohol	1.0 [1.0–4.0]	
	Cannabis	1.0 [1.0–1.0]	
	Co-Poisoning	2.0 [2.0–18.0]	
	Aluminum phosphide	6.5 [3.0–6.5]	
	Pesticides	3.0 [2.0–5.5]	
	Others	2.0 [1.0–8.0]	
	Unknown	3.0 [2.0–7.25]	
Cause of poisoning	Casual	2.0 [1.75–9.0]	χ2 = 3.62 *P* = 0.30
	Suicide	2.0 [2.0–4.0]	
	Accidental/overdose	3.0 [2.0–5.5]	
	Unknown	3.0 [2.0–5.0]	
Route of use	Oral ingestion	3.0 [2.0–4.0]	χ2 = 1.84 *P* = 0.61
	Inhalation	3.0 [2.0–12.0]	
	Injection	3.0 [1.0–3.0]	
	Unknown	2.0 [1.0–5.0]	
Outcome	Survival	2.0 [2.0–4.0]	Z = 4.27 *p* < 0.001
	Non-survivors	5.5 [2.0–13.0]	

Mean time from exposure to hospital arrival was 6.96 ± 12.94 (Range: 0.5–72) hours. Results of Kruskal Wallis test showed that there was a significant difference for time between exposure and presentation to hospital when comparing various xenobiotics [Opioids 9.18 ± 17.35 h, Alcohol 14.37 ± 22.67 h]. Mann-Whitney test showed that those exposure to alcohol and unknown agents had higher time of presentation to the hospital, in comparison with other agents (*p* = 0.03; Table 6).

**Table 6:** Mean time from exposure to poison and arriving to hospital in various poison agents

Poisoning agent	Hours (Mean ± SD)	Median[IQR]	Test result
Acetaminophen	2.00 ± 0.00	2.00[2.00–2.00]	Χ2 = 5.17 *P* = 0.03
Tramadol	5.30 ± 4.46	4.00[1.25–10.00]	
Benzodiazepines	4.97 ± 4.12	3.00[2.00–10.00]	
Tricyclic antidepressants	3.54 ± 3.53	2.00[1.00–5.00]	
Antipsychotics	2.78 ± 2.62	2.00[0.75–4.50]	
Anticonvulsant	2.35 ± 2.12	2.00[2.00–2.00]	
Beta Blocker	3.00 ± 0.89	3.00[2.00–4.00]	
Opioids	9.18 ± 17.35	4.00[1.00–10.00]	
Alcohol	14.37 ± 22.67	4.50[0.62–38.00]	
Cannabis	2.98 ± 2.35	2.00[1.50–3.00]	
Co-Poisoning	5.00 ± 4.24	5.00[2.00–7.00]	
Aluminum phosphide	5.50 ± 0.71	5.00[5.00–5.00]	
Pesticides	9.04 ± 19.31	2.00[1.25–9.50]	
Others	2.00 ± 1.73	1.00[1.00–1.00]	
Unknown	19.00 ± 22.58	6.00[3.00–48.00]	

## Discussion

Poisoning remains as a significant medical complaint across the globe [8, 12–20] with various patterns of acute toxicity in different regions. South Khorasan province especially Birjand city in Iran is no exception, and there is a paucity of information regarding poisonings in this region. According to our results, the most common agents of poisoning were pharmaceutical drugs, opioids followed by pesticides.

Pharmaceutical drugs were the most commonly used group of toxic substances in our patients, which is consistent with previous studies in Iran and other countries [12, 17]. This observation can be attributed to the accessibility of pharmaceutical drugs in Iran, especially in the studied region. We suspect that the availability of benzodiazapines without prescription is a major contributor to its common use amongst this population.

Opioids poisoning was the second most common agent of poisoning in this study. Several recent studies have shown that opioids poisoning is the most common cause of death annually in many regions of Iran. For instance, Farzaneh et al.[18] in the northwestern city of Ardabil, Afzali [19] in Hamadan City (west of Iran), and Ayatollahi et al. [20] in Yazd City (center of Iran) illustrated that from among xenobiotics, opioids were the most frequent cause of acute poisoning. In Iran opium and opium residue are the most common opioid. Infact Iran has the highest rate of opium addiction in the world [21]. The availability of opioids in the region due to the sharing border with Afghanistan and the sociocultural characteristics of Iran are potential contributory factors to this finding. The high rate of opioid poisoning is similar to other geographical areas as evidenced by Hamilton et al. [22] who reported that the opioids and other medications are the most common causes of acute poisoning in New York City.

Pesticides were found to be the third most common agent of poisoning. Azin et al. (2008) conducted a study in six big cities of Iran and reported similar results [23]. Most recently, poisoning with AlP is the cause of many poisoning-related deaths in Iran for which no antidotes have yet been reported [24, 25]. Mortality rate from AlP poisoning is even high in the ICU patients, and in some studies, it has been reported to be 75–100% [26]. In our study, two (0.7%) cases of AlP poisoning, both severe cases, were recorded but improved by means of certain treatments including intra-aortic balloon pump insertion. We have already reported successfully treated cases of severe AlP poisoning by this method [27].

Interestingly, three (1.4%) of the patients in our study used a compound locally called *Majoon Birjandi*, which is unique to this region of Iran [21]. It is a mixture of several hot-natured herbs mixed with some cannabis. Made in solid form in diamond-shaped molds, it is used mostly by young people to induce euphoria. No mortality in such cases was observed.

The frequency distribution of consciousness level at referral based on Glasgow Coma Scale/Score (GCS) showed that most patients had a score of than 9. In the study of Taghaddosinejad et al. [28] in Baharlou Hospital in Tehran, the frequencies of the REED coma grades 2, 3, and 4 were 49.7, 41.7, and 8.6%, respectively.

In our study, 12.4% of the patients developed seizure after poisoning, while in the study of Taghaddosinejad et al. [28] in the poisoned patients in the intensive care unit (ICU), 2% of the patients developed seizure, which was associated with the agents of poisoning, especially tramadol. In our study, seizures occurring in patients without a seizure history were related to tramadol poisoning. The relationship between tramadol poisoning and seizure has been reported [29, 30].

In our study, 41.2% of patients were intubated. This similar to Sulaj et al.’s study (2015) where 31.4% of patients underwent mechanical ventilation[31]. In the study of Ahuji et al. (2015) on 67 patients, 43 (64%) needed mechanical ventilation [32], and in Lam et al.’s study in Hong Kong, 67.9% of 265 patients were intubated [33]. Much lower intubation rates in acute poisoning patients have been reported. For example, in the studies of Exiara in Greece [34] and Ismail Demirel in Turkey [14], 4.48 and 6.2% of the patients were respectively intubated because of respiratory failure.

Mortality rate in our study was 19.5%. Similarly, Khodabandeh et al. [35] reported an approximately 27% mortality rate in the Tehran-based Loghman-Hakim Hospital ICU.

In our study, opioids and pesticides were the main causes of poisoning-related death. The overall mortality rate in the ICU in the Iranian-based hospital poison centers in Tehran, Khoramabad, Mazandaran, and are reported as 17.7, 11.6, and 14.6% respectively [36, 37].

This mortality rate is considerably high and should be therefore be thoroughly examined. In previous studies in Iran, similar rates of mortality have been reported in the ICU poisoned patients [11.6–18.6%) [38, 39]. Similar mortality rates have been reported in other developing countries [3, 40]. However, two recent studies in Germany and Hong Kong have reported mortality rates of, 0.7% and 3% respectively in the ICU poisoned patients [33, 41]. Several reasons can be presented to explain the high variation in mortality rates in different countries. One possibility is that a referral center for poisoning, as with our study setting, may have selectivity toward more severe poisonings leading to admission. Another possibility is that the criteria for admission to the ICU are widely different in different hospitals and countries [33]. Moreover, in our setting, ICU admission was limited only to obviously severe and life-threatening poisonings, while other institutions routinely admit all poisoned patients to the ICU, irrespective of the severity of their symptoms at arrival [39].

Interestingly, most of the mortality in our center was related to opioid and unknown cases. In a 6-year study in Tehran, Hassanian-Moghaddam et al. [42] reported that opioids were the most common cause of poisoning and second most common cause of death after pesticides. In another study, Torkashvand et al. [38] illustrated that 23.8 and 8.1% of the 260 poisoned cases were caused by methadone and opium, respectively. Similarly, in the study of Bjornaas [17] in Norway, opioids (48.1%) were the most common agent of poisonings leading to death . In the study of Shadnia in Tehran, Iran, opioids (40%) were reported as the most common cause of death [7]. It seems that since opioids can cause hypoxia, delay in treatment of toxicity may cause brain hypoxia and also death. Addressing opioid toxicity promptly with reversal agents and/or mechanical ventilation and save thousands of lives. Take-home naloxone programs are expanding around the world and may be applicable to patients in our study area. In these programs, those who abuse drugs, especially those addicted to opioids and their relatives are given naloxone to be administered subcutaneously or intranasally by bystanders or by the individual user after opioid overdose.

Our study had some limitations. We were unable to determine mortality rate and outcomes in poisoned patients referred to the emergency department. Therefore, we could not determine the in-hospital mortality rate in all the poisoned patients. The external validity of our study is also limited to the data from a single center, although the studied center is the main referral hospital for poisoning in the province and one of the main centers of this type in eastern Iran. Another limitation was its retrospective design. Because of the retrospective nature of this study, it is possible that some information is missed. Moreover, no data were available concerning the outcomes after hospital discharge, and death occurring after discharge may have been specifically missed.

## Conclusion

In our study, the most common agents of poisoning were pharmaceutical drugs, opioids followed by pesticides; most cases were suicide attempts and the mortality rate associated with the study population was high. This study was the first study evaluating cause and pattern of poisoning cases in the South Khorasan Province in east Iran. This study has data that compliments the literature and can help to characterize the poisoning cases seen in this area.

Opioid poisoning is a major concern and most important cause of death due to poisoning in the region. Recognition of this important cause of death may lead to actions to mitigate harm such as providing naloxone for potential opioid misusers. Self-administration of naloxone can decrease time to treatment after overdose and reverse life threating toxicity before it is too late. Further measures to address poisonings, such as regulation to deal with drug trafficking and laws controlling the availability of these substances can help reduce cases of these preventable causes of death.

## Abbreviations

AlP: Aluminum phosphide
CO: Carbon monoxide
ED: Emergency department
HIS: Hospital information system
ICD10: International classification of diseases, tenth revision
ICU: Intensive care unit
RR: Respiratory distress
